# Retraction Note: Comparison of bone morphogenetic protein and autologous grafting in the treatment of limb long bone nonunion: a systematic review and meta-analysis

**DOI:** 10.1186/s13018-021-02491-6

**Published:** 2021-05-29

**Authors:** Yong-Qiang Zhou, Hong-Liang Tu, Yan-Ji Duan, Xiao Chen

**Affiliations:** 1 The Department of Orthopedic Surgery, The First People’s Hospital of Neijiang, Neijiang, 641000 Sichuan China; 2 The Department of Neonatology, The First People’s Hospital of Neijiang, Neijiang, 641000 Sichuan China

**Retraction note to: J Orthop Surg Res 15, 288 (2020)**

**https://doi.org/10.1186/s13018-020-01805-4**

The Editors have retracted this article because it contains material that substantially overlaps with content from another article by different authors.

All authors agree to this retraction.

